# The Pathology of Inflammation

**Published:** 1896-01

**Authors:** Geo. E. Hunt

**Affiliations:** Indianapolis, Ind.


### THE DENTAL REGISTER.

VOL. L.] JANUARY, 1896. [No. 1.

### COMMUNICATIONS.

### The Pathology of Inflammation.

BY GEO. E. HUNT, D.D.S., INDIANAPOLIS, IND.

Read before the Tri State Dental Meeting, June 15th, 1895.

For purposes of description the process of inflammation may be divided into—First: Changes in the blood-vessels and circulation. Second: Exudation of fluid and of blood corpuscles from the vessels. Third: Changes in the inflamed tissues. This division is made merely to facilitate consideration of the subject and it must not be imagined that these changes occur successively, for such is not the case; they are all taking place at the same time.

The changes occurring in the blood-vessels and circulation are marked. We have first dilation of the vessels in the affected area, with increased rapidity of blood flow. Dilatation occurs primarily in the elastic arterioles. The veins ranking next in elasticity are soon proportionally distended, while the non-elastic capillaries are dilated but little.

Irritation of a sensory nerve will produce dilation of the arterioles in the area to which the nerve is distributed, by reflex action. The arterioles being dilated and the blood pressure remaining the same, a larger quantity of blood will pass through them. On account of their non-elastic walls the capillaries connecting with these arterioles do not dilate proportionally; consequently, blood pressure in them is increased and acceleration of blood flow through them results.

After about an hour of this accelerated blood flow, the next stage, that of retardation of blood flow, is reached, the vessels continuing dilated. Retardation is due to certain changes in the inner or endothelial wall of the smaller veins, which cause it to become sticky. This degenerative change also affects the cement substance binding together the edges of the endothelial cells constituting the vessel wall, deteriorating it and rendering it more penetrable.

The increased friction produced by this sticky condition of the vessel wall is soon evidenced by the gradual flowing of the blood current and the tendency of the leucocytes to adhere as they roll and tumble along the periphery of the lumen. It is probable that this morbid condition embraces also the inner wall of the arterioles, inasmuch as it is histologically identical with the corresponding wall in the veins, but the adhesion of leucocytes is greater in the veins than in the arteries because the blood is not driven in successive waves in the former and the flow is not so intermittingly swift. The valves in the veins also assist in causing arrest of the leucocytes. The red corpuscles, being heavier, are swept along in the axial stream and are not yet affected.

Some of the white corpuscles will stick for a moment, but will be released by the flow of the current and will pass on through the irritated region to the normal tissue beyond. Others will be arrested and remain sticking to the vessel wall. Still others will roll up against these adherent ones and before they can work their way around will be caught and retained, and in a short time the wall of the veins in the affected region will be found lined with adherent leucocytes. Other corpuscles adhere to these, and the lumen of the vein is gradually occluded—the blood current becoming proportionally slower. This process extends back into the capillaries also.

As long as there is a channel of sufficient caliber in the veins the red corpuscles pass on through, but when that is closed to them, or so nearly so that only a few may find a way, the others mass themselves in the capillaries and arterioles, causing these latter vessels to look as though filled with a red injection mass.

There are a few leucocytes mixed with the red corpuscles, about the usual proportion as found in the normal blood.

But before the mass of red corpuscles in the capillaries and arterioles become too great to prohibit it, we have the stage of oscillation in these vessels; this occurs when the veins have so nearly occluded that the flow is materially arrested. Then, at each heart impulse, the blood surges into the arterioles and capillaries leading to the affected veins, and not being able to pass through, flows back during diastole. Systole sends another wave up to the affected region which again recedes when the force of the heart beat has expended itself.

Following oscillation is the natural sequence of this progressive occlusion—stasis. In stasis the blood mass is stationary in the vessels, although it may remain fluid for two or three days. During stasis the capillary wall, being unnourished, gradually perishes—starves to death. When this occurs thrombosis or coagulation of the blood takes place.

This, in brief, constitutes the changes occurring in the blood and circulation.

Coincident with dilation and increased blood flow, we find the normal exudation of fluid from the vessels much increased in quantity and changed in quality—becoming much more albuminous and consequently more coagulable. At first the lymphatics, by extra efforts, are able to remove it, but in a short time it is poured out in such quantities that those vessels are overtaxed and the fluid accumulates in the connective tissue spaces, swelling the parts.

Now, if a small vein or capillary is closely observed, leucocytes are seen passing through the vessel wall, at first scatteringly, but eventually in great numbers. They pass out through small openings in the cement substance which joins together the edges of the endothelial plates constituting the vessel wall. During inflammation, while the vessels are distended, this cement substance gives way in the shape of minute holes or stigma, which gradually become larger and are known as stomata.

The leucocytes pass out through the stomata mainly by virtue of their amœboid movements. At first a small, button-like elevation may be seen on the outside of the vessel wall. This gradually increases in size as the substance of the leucocyte flows through until the cell will be pear-shaped with the small end adherent in the hole in the cement. This little pedicle finally comes away and the passage is complete. The cause of this diapedesis of leucocytes may be found primarily in the deteriorated condition of the vessel wall, by which facilities are afforded for its penetration, and in the inherent tendency of the cell to exert its power of amœboid motion. Contributory conditions exist in the pressure of fluid and other leucocytes from within and the probable fact that the surface of the endothelial plates are sticky and the cement substance, through which the cells pass, is not, so that passage through the stoma is easier than passage along the inner surface of the wall over the sticky endothelial plates.

The red corpuscles, not being endowed with amœboid motion, are not found in any quantity outside of the vessel unless death or rupture of the vessel wall occurs; then an immense diapedesis of red corpuscles may occur from the engorged vessels. A few red corpuscles are seen in the earlier stages, but they are those that some unwonted circumstance has thrown in front of a large stoma, and which the blood current has forced through.

The white and red corpuscles thus set free in the tissues are washed along by escaping fluid, and crowded forward by other leucocytes escaping, and finally may wander some distance from the vessel from which they escaped. The leucocytes also move away by their power of amœboid motion. Thrombosis, when it occurs, puts an end to the escape of fluid and of corpuscles.

The changes occurring in the inflamed tissue may be briefly described. The tissue is softer than natural, usually watery-looking, blurred, and the cells indistinguishable. The cells separated by fluid and obstructed by fibrine filaments and leucocytes, nourishment ceases and necrosis may occur.

Such, in brief, is the course of all inflammations. One point must, however, be borne in mind. If the cause of the irritation is removed and the general health is fair, an inflammation may be stopped at any stage. It may proceed to the point of dilation with increased flow only. That is hyperæmia and a well known example may be found in the red hands occurring from handling snow or ice, or the redness of the face from exposure to heat; or, it may pass on to decreased flow, oscillation, or even stasis, and yet re-solution occur without loss of substance. If coagulation takes place, an abscess with loss of tissue is bound to result.

Redness, heat, pain, swelling and impaired function—the clinical signs of an inflammation—are thus accounted for. Redness and heat are directly dependent on the amount of arterial blood passing through the part in a given time. In the earlier stages of the inflammation, during dilation with increased flow, there is an abnormal quantity of blood, fresh from the heat-producing centers of the body, passing through the diseased part. If that part lies superficially, redness and increased heat are markedly present. But if the inflammation continues to stasis, the inflamed area becomes colder than normal and is either bluish or motley colored. Inflammations of the internal organs are not accompanied with increased heat in the part. An inflamed area of lung is never hotter than normal lung, and in the later stages of inflammation will be colder; rise in temperature, when it does occur, is due simply to more rapid circulation of the arterial blood. The lungs, being already at the temperature of arterial blood, cannot become hotter. There is no heat generated in an inflamed area. Increased nutritive movements are required to produce heat; but decrease of functional activity, degeneration and death are accompanied by a corresponding depression of temperature.

Swelling is due to the exudation of fluids and corpuscles into the part; pain, to pressure of such exudate on the terminal nerve endings, mainly. Impaired function is the result of the general injury to the tissues.

Inflammations are divided by the pathologists into serous, fibrinous, productive and suppurative. The first two are but steps leading to one of the later forms of inflammation. Serous inflammation is due to a slight injury, usually. The exuded fluid is a little more albuminous than normal, and is increased in quantity, but there are few, if any, leucocytes in it. In this form there is but little tendency to coagulation of the exudate.

If the irritation persists, the percentage of albumen, fibrinogen and white corpuscles is raised, and fibrin flakes form in various parts of the tissue. This stage is known as the serofibrinous form and leads on to the next, or fibrinous form of inflammation, in which the exudate is more richly albuminous and contains more leucocytes. There is a greater tendency to coagulate and "lymph" forms in the inflamed area. This lymph consists of fibrin containing leucocytes in its meshes and is typified by the exudate which glazes over the open wound a few hours after its infliction.

From the fibrinous stage an inflammation may become productive. Productive inflammations are those in which new tissue is formed; and without productive inflammation repair of wounds and restoration of lost tissue could not take place.

For the repair of lost tissue it is necessary that the causative agent be removed and that the parts be kept clean. If the loss of tissue be the result of traumatic influences, asepsis alone is all that is necessary; if tissue loss be caused by suppuration, removal of the irritant and the asepsis are indicated.

Productive inflammation is most conveniently studied in a gaping wound or a healing ulcer, because in these we may see the process as it occurs. The changes are precisely the same in the filling in of the space left after the evacuation of an abscess, but for purposes of description, the healing of an ulcer will be considered. The glairy, mucous-like lymph, which glazes the bottom and sides of the wound, becomes closely packed with leucocytes which have previously exuded from adjoining capillaries. Into this leucocyte-crowded matrix, spring little fibrillous capillaries—off-shoots of neighboring vessels—which, after penetrating the lymph to the free surface of the wound, form a loop, turning back into the lymph, and an anastomosing with either the parent vessel, or perhaps another new capillary. The leucocytes quickly gather round these capillary loops, for it is from the blood circulating in them that sustenance for this new tissue must be derived. This tissue is now known as granulation tissue, because of the granular feeling of these capillary loops when the finger tips are passed over them.

More lymph is now formed by the exudate from these new capillaries. This in turn becomes crowded with the leucocytes, and is penetrated by still newer capillaries, and the process is thus continued until the lost tissue is restored. The leucocytes develop into fibrous cells, and the new tissue thus formed becomes fibrous connective tissue. It is popularly known as inflammatory or scar tissue. Scar tissue is an inferior grade of tissue. It is always more susceptible to instant effects, and is prone to break down under influences that would cause but a trifling disturbance to the normal tissue which it has displaced.

If the action of the irritant is intense and prolonged, suppurative, instead of productive inflammation may follow the fibrinous form. Suppurative inflammation is largely due to the presence in and action on the tissues of certain bacteria, most commonly the staphylococus pyogenes aureous, or golden pus-producing cluster cocci. A form of suppurative inflammation may be induced by the action of certain chemical irritants injected into the tissue. The pus resulting from this irritation is free from bacteria, bland, and non-infectious. Pus resulting from the presence of bacteria is infectious, poisonous, and has a characteristic offensive odor.

For bacteria to propogate and perform their metabolic changes, it is necessary that the conditions be suitable. In the depressed functional activity, the lowered physiological resistance in tissue undergoing a severe inflammation, they find a suitable field for their life work. During metabolism, their waste products, or ptomains, are freely given off, dissolving and breaking down all tissue with which they come in contact. The cocci multiply rapidly and penetrate the tissues in all directions. Many of them die in their own effluvia, but propogation is so rapid that they are not missed.

After a few hours a layer of leucocytes may be seen surrounding the center of infection. Nature is endeavoring to repair the ravages of the micrococci by the formation of granulation tissue. At first the effort is in vain, the ptomains breaking down tissue in all directions, into a thin poisonous liquid. This liquid contains disintegrated tissue infiltrated with dead and dying cocci and leucocytes, and is known as pus. Outside of this purulent formation is an area where the cocci and leucocytes are almost equally divided, and the farther from the seat of infection, the fewer cocci are found.

If this condition occurs in the depths of tissues, it is known as an abscess; if on the surface, an ulcer. If an abscess, the dissolution of tissue takes place most readily in the direction of the free surface, that being the direction of least density and resistance, and the abscess, unless opened artificially, will eventually reach the surface and burst. When that occurs, or when from any cause the pus is evacuated and cleanliness maintained, the balance of power between the cocci and leucocytes is in favor of the latter, and repair by the formation of scar tissue, as previously outlined, begins. The process just described is that occurring in acute abscess.

If in suppurative inflammation, the irritation is of small degree, but persistent—if the depression of vital activity and consequent lowering of physiological resistance is limited in severity, but not in duration, a chronic instead of an active abscess may result. For this to occur, it is necessary that the action of the cocci be restricted. This may be from various causes. In the alveolar process, it may be due to non-exposure of the devitalized pulp to air, in which case mummification or dry gangrene of the pulp may occur, or it may be due to partial drainage of the abscess through the crown end of the root, relieving pressure, which latter is a potent factor in the burrowing of pus through tissue, or there may be a fistula opening from the abscess into the mouth, acting as a drain. In this latter case, formation of pus may take place so slowly that the mucous membrane around the opening of the fistula will heal after evacuation, and not require puncturing for days or weeks, until the accumulation of pus puffs it out in a tumor-like formation on the gum. But from whatever cause, if the action of the ptomains be so divested of virulency that the leucocytes are able to hold their own in the struggle for supremacy, a wall of granulation tissue will be formed about the focus of infection.

The action of the ptomains on the free surface of this wall will be intermittingly successful. When the vitality of the whole system is lowered by exposure, overwork, a cold, or any of various other causes, the equilibrium established between the action of the micrococci and that of the leucocytes is disturbed in favor of the former and the tooth, if it be an alveolar abscess, becomes sore. Often a little rest and tonic treatment with judicious stimulation of the gum over the affected part will restore the lost equilibrium and the tooth becomes easy once more.

In the course of months this wall of granulation tissue develops into fibrous connective or scar tissue, precisely as though the abscess had been evacuated and the entire lost tissue restored. This wall or sac is nature's effort to prevent the encroachment of pus on the neighboring healthy tissue. Now, if no new source of irritation arise, the fluid portions of the contents of this sac may be absorbed, leaving behind a dry, cheesy-looking mass that may remain encapsuled for years without any trouble, but eventually the conditions there will become favorable to the rapid propogation of bacteria and active inflammation will result. In case of an alveolar abscess, this variation in condition is often the result of decay reaching the pulp chamber and admitting moisture and bacteria to a dry, gangrenous pulp. Moisture and bacteria will quickly set up putrefaction, and all of the evils attendant on an active abscess will follow. This may also occur from mechanically opening up a long-since dead pulp without the necessary antiseptic precautions.

One thorough draining and cleansing of a small, active abscess is usually all the treatment necessary to effect a cure. But this is difficult to accomplish in the case of an alveolar abscess unless there is a fistula present. Given a fistula, and drainage is provided for. To thoroughly drain an abscess in the superior maxilla, through the root of a tooth at one sitting, is possible only under the most favorable circumstances. These favorable circumstances would be an abscess directly above the point of the root and a free opening through the latter. If the abscess is down on the side of the root, the operation is much more complicated. Abscesses in the inferior maxilla can seldom be thoroughly drained through a tooth-root at one sitting.

Therefore, considering the matter from the standpoint of the conditions present in the alveolus, immediate root filling is contra-indicated in these cases. It is a poor surgeon that would sew up a wound without providing for drainage.

But in chronic abscesses the contra-indications are still stronger. In this case, in the absence of a fistula, we have not only the same difficulty to contend with that we had in the acute abscess, namely, imperfect drainage, but the case is still further complicated by the probable presence of this "pus sac" of fibrous connective tissue. A plentiful formation of blood-vessels is absolutely necessary to the production of scar tissue. A newly-healed wound is always red, indicating an extensive blood supply. But in the course of months it becomes white. The fibrous connective tissue has contracted; these numerous capillaries, so useful in building up the new tissue, have fulfilled their purpose, and are now dispensed with, an old scar tissue has even fewer blood-vessels than had the normal tissue which it has replaced. Any one who will carefully examine a "pus sac" on the root of a tooth will be struck with its resemblance to old scar tissue. The resemblance need be close for they are pathologically identical. In the constant struggle for supremacy between health and disease, during the existence of the abscess, this sac wall has passed through the granular stage, and has become scar tissue.

For the lost tissue in that locality to become entirely replaced with scar tissue, after removal of the irritant and evacuation of the abscess, it is necessary that this sac be either destroyed or absorbed so that an active inflammation—a productive inflammation—may be set up in the contiguous tissue. This, and this only, will promote a restoration of tissue. That it is practically impossible to do this in one treatment, in the absence of a fistula, will have to be conceded. If this abscess is drained and cleansed daily for a few days, judicious irritation of its walls may result in setting up inflammation enough for the production of new tissue. A good opening through the root and as direct access to the abscess as can be obtained are essential to success. These cases often baffle our utmost efforts to control them. If there is a fistula present, the matter is much simplified. The root may be filled and the fistula will serve not only as a drain, but as a channel of medication, if any such is needed after one thorough treatment.

Therefore, it may be assumed that the presence of an abscess, where acute or chronic, is a contra-indication to immediate root-filling unless there is a fistula leading from the point of infection. To obtain the best results, drainage must be thorough. That is a simple surgical axiom. And without a fistula, the root-canal must be depended upon for drainage.

This view of the question totally ignores the arguments for and against immediate root-filling that have for their base the conditions present in the pulp-chamber and canal. That is another matter. But from the standpoint of good common-sense surgery, and in the light of our knowledge of the pathology of these cases as just outlined, condemnation of immediate root-filling cannot but occur.

The immediate root-fillers have been short-sighted. They argue entirely from the condition in which they get and leave the tooth. More important, because more uncertain, questions are the condition of the peridental membrane and the adjoining alveolar process.

DISCUSSION BY E. S. HOLMES, GRAND RAPIDS, MICH.

Mr. President: It is fortunate, on account of the crowded condition of our programme, that the subject treated in this paper and the lucid manner of its presentation renders a lengthy discussion unnecessary. I shall, therefore, occupy your time but a few minutes.

In his enumeration of the histological elements affected by inflammation, one tissue that seems to me to play quite an important role in this lesion is omitted by our essayist, probably inadvertently. I refer to the "third corpuscle" of the blood. Prof. Senn's Principles of Surgery, page 72. The slowing up of the blood flow in certain stages of inflammatory action, and the degenerative changes in the endothelial wall of the smaller veins causing it to become "sticky," are given by the author as the direct cause of the retardation of the leucocytes that in abnormal conditions flow at the periphery of the lumen, or on the outside of the blood current in the vessels; whereas, it seems to me that the viscid condition of the interior wall of the vessels is not the direct cause of the retardation in the flow of the white-blood corpuscles, but that it is due to the "third corpuscles," which, being small and light, flow on the extreme outside of the current, this forming a rough floor on the periphery of the lumen on which the leucocytes roll and tumble along and are thus retarded. Bowl a ball on a smooth, clean pavement and it will go a long distance, but the same ball, bowled with the same force over the same pavement covered with gravel, will be very much retarded and soon stopped. Thus the third corpuscles being retarded or adhered to the inner wall of the vessel forms a rough floor over which the blood flows with increased difficulty on account of friction, which it seems to me is a very important cause of retardation and stasis.

In the interest of accuracy of expression, perhaps attention ought to be called to the word "diapedesis" where it first occurs in this paper. It is here used to express the flow of the white corpuscles through the walls of the vessels. Now as the leucocytes do not flow through, but "crawl" through by amœboid action, it seems to me to be an improper use of the word, and that it should be used, as it is farther on, to describe the flow of the non-amœbic corpuscles through the stomata in the walls of the vessels.

Permit me to refer to one other point in this paper where the essayist seems to take a position contrary to the generally received opinion. He says: "there is no heat generated in an inflamed area." One of the symptoms of inflammation is elevated temperature. If this increased heat is not produced "in an inflamed area," where is it generated? If in the lungs—the central furnace for supplying heat to the economy—then why is not the whole tract from the lungs to the "inflamed area" super-heated?

But as the temperature of the arteries is the same except in general fever, it may be claimed that the increase of the heat of the part is by accretion. But as the accretion is "in the inflamed area" it seems to me to be at least equivalent to the generation of heat at that point. The great source of heat is the chemical union of oxygen with carbonaceous material. The heat of your furnace is in exact proportion to the amount of oxygen combined with the fuel in it.

The normal temperature of all living organisms is kept up in obedience to the same law. The abnormal increase of temperature is produced and kept up by the excessive chemical union of oxygen and carbon, just as any other fire is kept up by combustion; and the pathological fire-place must be at the "inflamed area." The large consumption of carbonaceous material, by its union with oxygen, is the reason why inflammatory diseases are so very exhausting, both the elements being supplied by the blood.

The latter part of the paper being devoted to practice, founded on what seems to be correct principles, I have only to express my commendation and relieve your patience.

